# Challenges of aortic valve tissue culture – maintenance of viability and extracellular matrix in the pulsatile dynamic microphysiological system

**DOI:** 10.1186/s13036-023-00377-1

**Published:** 2023-09-28

**Authors:** Claudia Dittfeld, Maximilian Winkelkotte, Anna Scheer, Emmely Voigt, Florian Schmieder, Stephan Behrens, Anett Jannasch, Klaus Matschke, Frank Sonntag, Sems-Malte Tugtekin

**Affiliations:** 1 Department of Cardiac Surgery, Carl Gustav Carus Faculty of Medicine, Technische Universität Dresden, Heart Centre Dresden, Fetscherstr. 76, 01307 Dresden, Germany; 2 Fraunhofer Institute for Material and Beam Technology IWS, Dresden, Germany

**Keywords:** Calcific aortic valve disease, Tissue culture, Microphysiological system, Viability, ECM remodelling

## Abstract

**Background:**

Calcific aortic valve disease (CAVD) causes an increasing health burden in the 21^st^ century due to aging population. The complex pathophysiology remains to be understood to develop novel prevention and treatment strategies. Microphysiological systems (MPSs), also known as organ-on-chip or lab-on-a-chip systems, proved promising in bridging in vitro and in vivo approaches by applying integer AV tissue and modelling biomechanical microenvironment. This study introduces a novel MPS comprising different micropumps in conjunction with a tissue-incubation-chamber (TIC) for long-term porcine and human AV incubation (pAV, hAV).

**Results:**

Tissue cultures in two different MPS setups were compared and validated by a bimodal viability analysis and extracellular matrix transformation assessment. The MPS-TIC conjunction proved applicable for incubation periods of 14–26 days. An increased metabolic rate was detected for pulsatile dynamic MPS culture compared to static condition indicated by increased LDH intensity. ECM changes such as an increase of collagen fibre content in line with tissue contraction and mass reduction, also observed in early CAVD, were detected in MPS-TIC culture, as well as an increase of collagen fibre content. Glycosaminoglycans remained stable, no significant alterations of α-SMA or CD31 epitopes and no accumulation of calciumhydroxyapatite were observed after 14 days of incubation.

**Conclusions:**

The presented ex vivo MPS allows long-term AV tissue incubation and will be adopted for future investigation of CAVD pathophysiology, also implementing human tissues. The bimodal viability assessment and ECM analyses approve reliability of ex vivo CAVD investigation and comparability of parallel tissue segments with different treatment strategies regarding the AV (patho)physiology.

**Supplementary Information:**

The online version contains supplementary material available at 10.1186/s13036-023-00377-1.

## Background

**Fig. 1:**
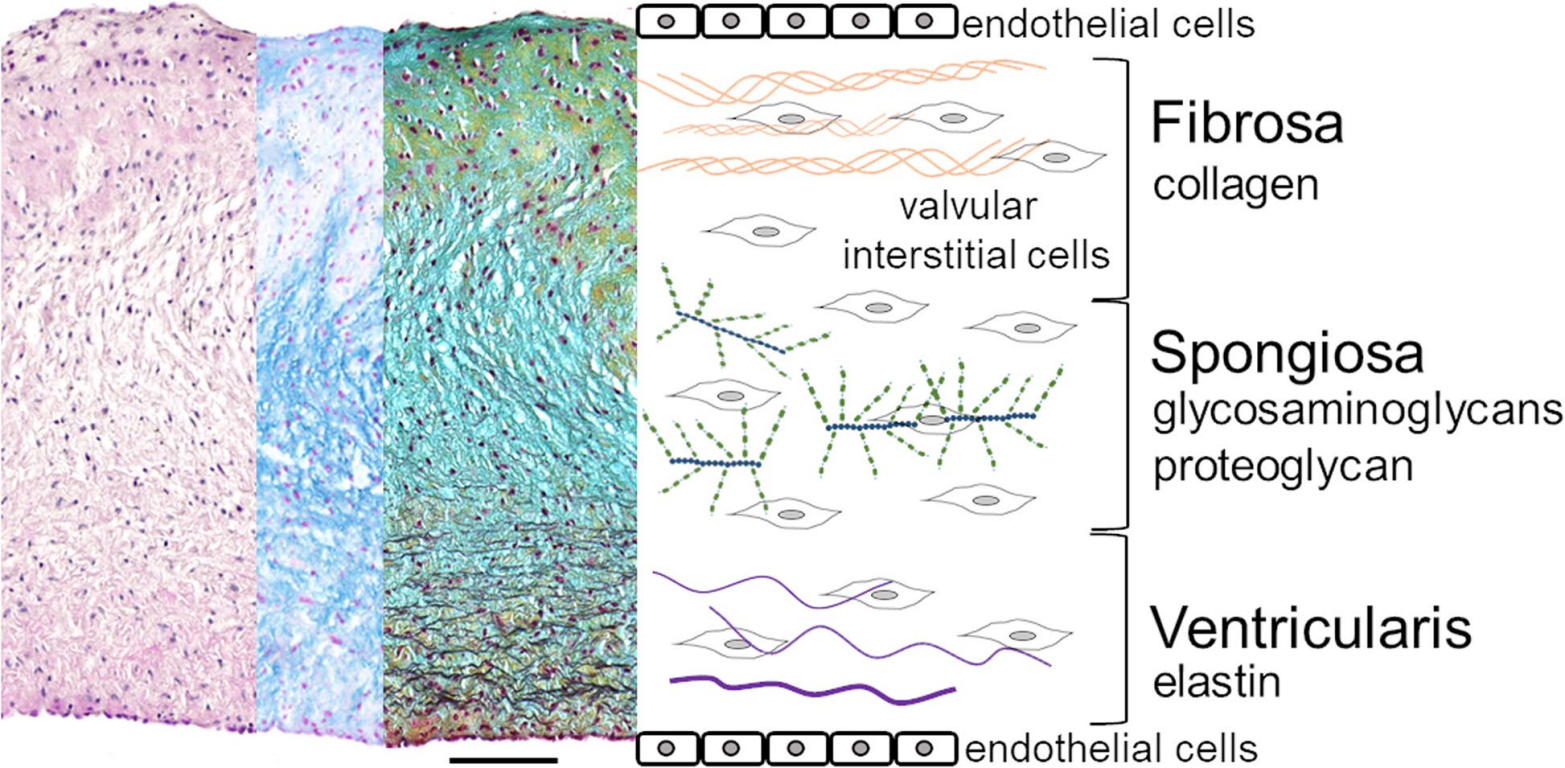
Three-layered aortic valve tissue in histology and schema: HE, Alcian blue and Movat Pentachrom stain illustrate dominating ECM components in fibrosa, spongiosa and ventricularis layer of aortic valve leaflet covered with endothelial cells. Valvular interstitial cells are responsible for ECM maintenance in non-diseased tissue (Scale bar: 100 µm)

The calcific aortic valve disease (CAVD) characterizes a degenerative obstruction of the aortic valve [1]. In 2019, 9.4 million patients worldwide were diagnosed with CAVD [2]. Conservative treatment options are not available and the only therapy remains surgical aortic valve replacement. The human aortic valve (hAV) consists of three distinct layers: the lamina ventricularis, the lamina spongiosa and the lamina fibrosa (Fig. 1). The calcification process most frequently initiates at the fibrosa side adjacent to the aorta [3, 4]. Valvular endothelial cells (VEC) are located at the surface of the valve, while valvular interstitial cells (VIC) are dispersed in extracellular matrix (ECM). VICs are responsible for tissue maintenance [5–8]. CAVD is caused by active processes in which VICs experience pathological differentiation to myofibroblasts or osteoblast-like cells. These modified VICs actively deposit bone substrate. In parallel, passive hydroxyapatite intercalation, e.g. after cellular apoptosis, in between collagen fibres augments pathogenesis [9–20].

2D-cell culture models of human and porcine VECs and VICs were conducted [9, 21–24]. Nonetheless, cell–cell communication, ECM, biomechanical and -chemical emulation were not represented physiologically [25]. Animal models are used but the in vivo approach held aberrant microanatomy, physiology and calcification potential that impede result interpretation and transferability to the human system [9, 15, 26–32]. Diverse ex vivo bioreactors were introduced to bridge in vitro and in vivo approaches by reflecting diverse microenvironmental parameters. 2D-tissue cultures in microphysiological systems (MPSs) were established to elucidate calcification pathways [24] including miRNAs [3] or to investigate the effect of hypoxic environment [33]. In succession, tissue culturing in MPSs was realized to model appropriate cell–cell and cell–matrix interaction as well as biomechanical and -chemical simulation. Tissue stretch, culture medium pressure, shear forces with side-specificity, varying oxygen supply and *dynamic* incubation of aortic valve cuttings or entire aortic roots were modelled and performed for incubation periods of up to 56 days (Table 1) [3, 4, 34–41]. The presented study introduces an innovative MPS consisting of a pneumatic pump chip in conjunction with a tissue incubation chamber (TIC). In contrast to previous bioreactors, this novel system allows incubation of integer porcine AV (pAV) and hAV tissue cuttings of intermediate scale. The applied tissue of only 15 mm^2^ allows several cuttings from the same AV to be incubated in parallel as technical replicates. In case of diseased AVs, mostly from human origin, the intermediate scaling provides the opportunity of choosing macroscopically healthy AV regions instead of applying the entire degenerated valve. VICs remain physiologically dispersed in ECM, VECs surround the leaflet and the MPS enables the application of adjustable biomechanical force to achieve physiological model parameters [3, 6, 19, 41–47]. The MPS allows a great parameter diversity and model adaption albeit construction and tissue implementation demand profound evaluation. Detailed ECM analyses and a bimodal viability assessment are introduced to investigate AV tissue transformation and survival following long-term incubation [33, 48–54].

**Table 1:** Diverse AV bioreactors intend to mimic biomechanical model properties

Bioreactor (model properties)	AV origin	Incubation [days]	Reference	Tissue viability/apoptosis
**Dynamic incubation**				
Stable and disturbed flow opposition	Porcine	14	*Fernandez Esmerats* [3]	*Apoptosis Tunel Staining (anti- miR-483 induced apoptosis by twofold compared with control)*
Infective endocarditis model	Porcine	1–2	*Lauten* [55]	*None*
Simulation of AV coaptation	Rattine	7	*Maeda* [44]	*None*
Side-specific oscillatory and laminar flow	Porcine	2	*Mongkoldhumrongkul* [42]	*None*
Pro-degenerative treatment	Ovine	7	*Niazy* [35]	*None*
Shear force, osteogenic medium	Porcine	2–7	*Rathan* [4]	*Apoptosis Tunel staining (no apoptosis in either the fibrosa or the ventricularis when exposed to oscillatory shear)*
Shear forces	Porcine	5	*Sucosky* [56]	*Cell fragmentation, apoptotic bodies in DAPI nuclear staining (no cell fragments or apoptotic bodies)*
Side-specific shear stress	Porcine	4	*Sun* [37]	*Cell fragmentation, apoptotic bodies in DAPI, Apoptosis Tunel staining (No cell fragments or apoptotic bodies were detected, but minor cellular apoptosis *via* TUNEL assay in both tissue groups)*
Pulsatile shear forces	Porcine	1–3	*Sun* [38]	*None*
Shear forces	Porcine	2	*Weston* [57]	*None*
**Static incubation**				
Cyclic stretch (10–20%)	Porcine	1–2	*Balachandran* [46]	*Anti-bromodeoxyuridine cell proliferation IHC, Apoptosis Tunel Staining (Cell proliferation and apoptosis increased in a cyclic-stretch magnitude-dependent manner)*
Pro-calcifying medium (Static incubation)	Porcine	14	*Chester* [58]	*Caspase 3 apoptosis IHC (absence of apoptosis after 14-days incubation in media alone; presence of caspase- 3 in the calcified regions demonstrating impact of apoptosis on calcification)*
Cyclic stretch and pressure	Porcine	2	*Thayer* [59]	*None*
Pro-degenerative and -calcifying media (Static incubation)	Ovine	56	*Weber* [41]	*LDH-membrane integrity assay (27/56d), no lysis control; (LDH levels not elevated)*
Cyclic pressure	Porcine	2	*Xing* [60]	*None*
Anti-myofibroblast, osteogenic medium (Static incubation)	Porcine	28	*Zabirnyk* [39]	*None*

## Material and methods

### Tissue

pAV tissue was received from the local abattoir and complies with all relevant national regulations and institutional policies. The AVs were obtained from six months old *Landrace* pigs. The valves were excised, washed in PBS (Dulbecco’s phosphate buffered saline; *gibco, USA)* and kept in ABAM [DMEM (*gibco, USA*) + 1% antibacterial/ -mycotic solution (*Sigma-Aldrich, USA*)]. hAVs were obtained in accordance with the tenets of the *Helsinki Declaration* and approved by an equivalent committee including informed consent (Ethikkommission der TU Dresden, EK429102015). The valves were obtained from the operating room following AV replacement, they were conveyed to the laboratory immediately, washed in PBS and kept in ABAM.

**Fig. 2:**
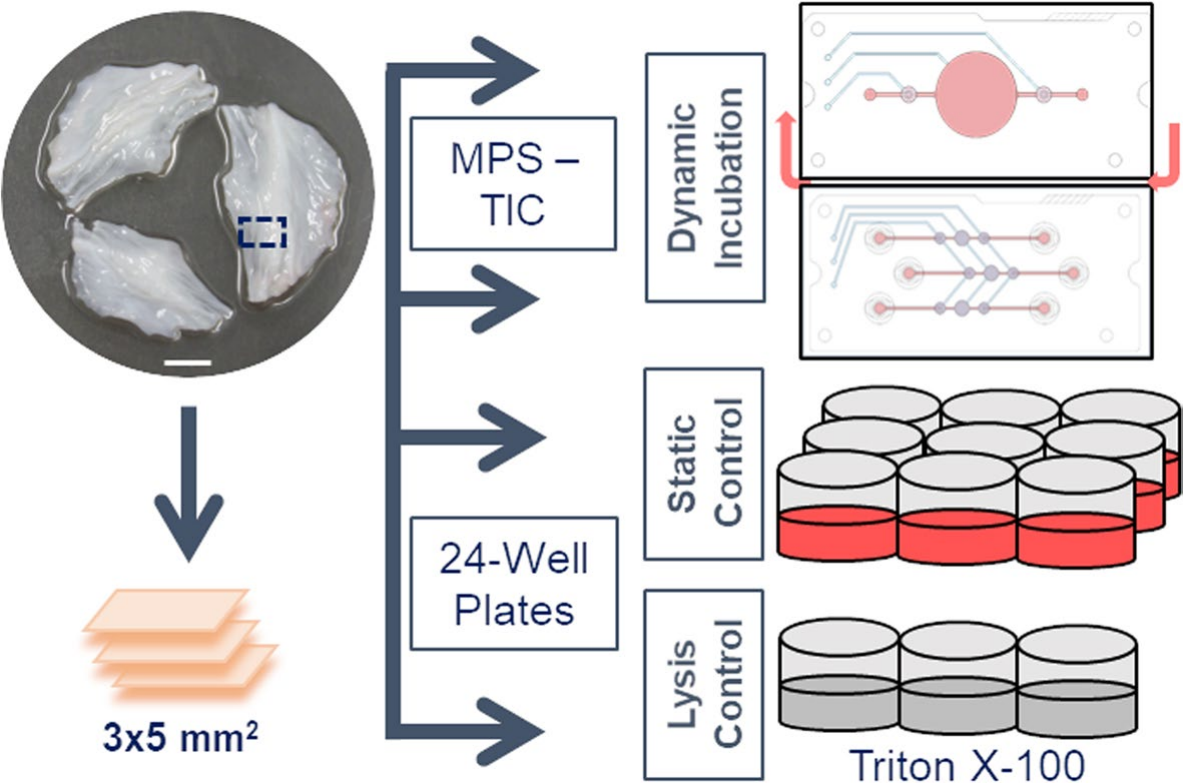
Experimental workflow TIC-MPS and control AV tissue culture: pAVs or hAVs (not shown) are excised, cut into 3 × 5 mm.^2^ specimens and deployed to *dynamic* setup using a *high flow* (upper chip layout) or *low flow* (lower chip setup), *static* or *lysis* control that are incubated in conventional 24-well culture plates (Scale bar: 5 mm)

pAV tissue subsections were used for model evaluation whereas hAV tissue sections were primarily used for applicability investigation. Age, disease and calcification level alterations in hAVs complicate model introduction. Left-, right- and non-coronary cusps were randomly allocated because of differences in collagen architecture [31]. Samples of 3 × 5 mm^2^ were excised from the coaptation margin of both pAVs and hAVs before subsections were scaled to 10 ± 0.5 mg (Fig. 2).

### MPS-TIC setup

**Fig. 3:**
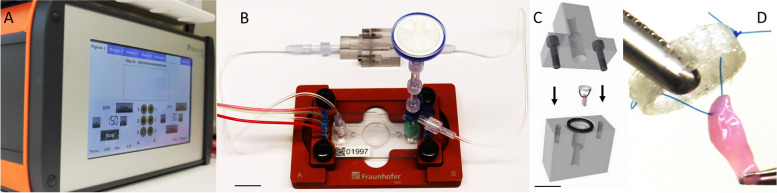
MPS-TIC Setup: **A** The MPS control unit enables regulation of pumping sequence and pneumatic pressure; **B** The high flow setup is conjoined with the attenuation element (here shown without culture medium); **C** The AV tissue specimen is stitched to a TPU ring and inserted into the tissue incubation chamber (TIC) (Arrows: flow direction [Q], scale bar: 1 cm). **D** Example of a stitched (TPU ring) pAV specimen prior insertion in the TIC

The MPS-TIC System is built up by interconnecting of the two functional modules TIC and pump chip. The TIC consists of two biocompatible polycarbonate (PC) shells of cylindrical excavation that constitute an incubation chamber with a diameter of 6 mm and a length of 20 mm. The tissue is integrated into the TIC with a 3D printed TPU-ring and the shells of the TIC are fluid tight connected with a sealing ring and two M3 screws. *Plug & play* modularity via LUER interfaces allows conjunction with adoptable pneumatic pump chips via common clinical tubes (*inner diameter: 1.2 mm; Braun, Germany*). The pump chips are assembled from fused PC layers comprising two functional compartments separated by a TPU membrane. The lower compartment contains the culture medium, valves and the pump chamber. The upper compartment contains pneumatic channels and interconnections to deflect the TPU membrane for pump actuation [48]. To run the pump, the chip is inserted in one of four support trays (*MPSbase, Fraunhofer IWS, Germany; *Fig. 3) which are connected to the control unit (*MPScontrol, Fraunhofer IWS, Germany; *Fig. 3)*.* Pulsatile pressure and vacuum alternation of + 250/ -150 mbar operates the two valves and the pump chamber granting directed culture medium propulsion [33, 48–50, 54].

Two pump chips are used to apply high and low flow within the TIC. A *low flow* setup with an established pump chip (pump chamber of 3 mm in diameter), was operated with a frequency of 150 bpm to achieve most physiological flow rates and shear forces [33, 50].

An up-scaled pump chip with a larger pump chamber of 20 mm in diameter and a pump frequency of 30 bpm was introduced for *high flow* AV incubation. Frequency increase was limited to 30 bpm, because the enlarged pump chip requires a longer period to fill with culture medium compared to the *low flow* setup. To decrease the pressure within the system, which is caused by the up-scaled pump chip, an attenuator element was implemented. The attenuation element also works as a bubble trap within the flow setup to maintain the system air-free.

#### Biomechanical computation

**Fig. 4:**
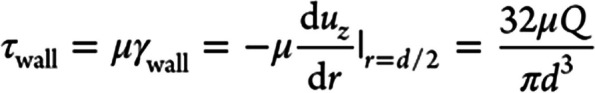
Shear force calculation: A Navier Stokes derivation is used to compute the shear forces inside a circular tube that act on the AV-tissue specimen (τ≙wall shear stress, μ≙dynamic viscosity, d≙diameter, Q≙ flow rate)

Micro-particle image velocimetry (PIV) was used to measure peak media flow velocity. Micro-particle suspension was propelled by the pneumatic pump chip through a channel of predefined dimension (3 × 0.25 mm2 intersection) and particle migration was tracked by high-speed *Complementary Metal-Oxide Semiconductor* camera in conjunction with an inverting microscope in the centreline of the channel [49, 61, 62]. Following, flow rates and shear forces according to the pulsatile flow character were inferred. To approximate shear forces acting on the AV tissue surface in a circular channel, a derivation of the *Navier–Stokes* equation has been applied (Fig. 4) [49, 63–65].

The small microchannel dimensions and slow culture medium velocities inhere low *Reynold* numbers. Hence, a laminar fluid character can be inferred [63]. The AV specimen is located at the centerline of the TIC, equally distributing culture medium flow (*Q*) on either side (Fig. 3B).

#### Experimental setup

The incubation period for pAV tissue sections in pulsatile dynamic vs. static culture was set to 14 days and four experimental arms were determined. hAV tissue specimens were incubated for 26 days to investigate sterility maintenance and tissue survival. DMEM (*Dulbecco’s modified eagle medium; gibco, USA)* containing 10% fetal calf serum (FCS; *gibco, USA*) and 1% penicillin (10000 IU)/ streptomycin (10 mg) (P/S; *Sigma-Aldrich, USA*) was used for AV tissue culture. Following experiment termination, the AV tissue mass was determined with a micro scale.

In the *dynamic* setups, AV tissues were sewed by *non-touch* technique to a TPU ring and inserted into the TIC (Fig. 3B) [33]. In *high flow dynamic* setup, the TIC was connected to the novel, upscaled modular MPS pneumatic pump chip providing pulsatile flow rates of 77.4µl/s at 30 bpm. 2.2 ml of culture medium were infused for incubation. The additional volume resided inside the attenuation element. In *low flow dynamic* setup, a common MPS pneumatic pump chip was connected to the TIC and allowed *dynamic* incubation at pulsatile flow rates of 13.4 µl/s at 150 bpm [33, 48, 49, 54]. 2 ml of culture medium were applied for incubation.

The *static* control AV specimens were placed in 24 well plates with 2 ml of DMEM culture medium each. *Lysis* control samples were incubated in DMEM with 10% FCS, 1% P/S and 0.25% Triton X-100 (*Serva, Germany)* solution throughout the entire incubation period.

#### Bimodal viability assay

AV tissue viability was assessed by two independent viability assays, a non-terminating resazurin reduction assay (RR assay) on the one hand and a histological lactate dehydrogenase (LDH) -viability stain on the other hand.

The RR assay is based on cellular uptake of non-fluorescent resazurin which is consecutively reduced to the fluorescent dye resorufin within viable cells [50, 66–70]. It was conducted at the beginning of the pAV experiment and on days 4, 8, 10, 12 and 14 for all four experimental setups. Prior to the measurement, both *dynamic* and *static* setup AV tissue specimens were transferred to a new 24 well plate and rinsed with ABAM. Two millilitres of DMEM–resazurin solution (300 µM) were added (Resazurin Sodium Salt; *Stemcell, Canada*). After 2 h of incubation, the medium was transferred and 100 µl were used for fluorescence assessment (*Tecan Infinite 200 Pro plate reader*; λ_exc._ 535 nm, λ_emm._ 590 nm). The measured arbitrary fluorescence units (FU) were relativized against culture medium control. Rate of viability was inferred by applying initial average FU results of each AV donor as reference. The RR assay was conducted according to preliminary validation measures [33, 50]. Every second day, the TIC and MPS components were disinfected with 90% ethanol and *static* comparison 24 well plates were exchanged to eradicate potential contaminations and prevent cellular consolidation. The resazurin reduction assay was equally applied every second day to evaluate hAV tissue survival for 26 days.

To proof substrate penetration, native pAV specimens were snap-frozen following the resazurin incubation period, cryo-sectioned and immunofluorescence (λ_exc._ 538–562 nm, λ_emm._ 570–640 nm) of the assay product resorufin was depicted. Average penetration depth was computed by using three representative circumferentially located AV regions in each pAV tissue subsection (*ImageJ Software 1.8; n* = *3).*

The LDH-stain is based on tetrazolium salt conversion to blue-coloured formazan by viable cells. The result designates site-specific tissue viability. Following 14 days of incubation, pAV tissue specimens were embedded in O.C.T. (*Tissue-Tek Sakura, USA*) and snap frozen before being cryosectioned (12 µm). The staining procedure described by *Jähn and Stoddart* was adapted to AV tissue properties [33, 52, 53]. To obtain two millilitres of staining solution, 0.53 mg Gly-Gly buffer (*Sigma-Aldrich, USA*), 1341 mg Polypep (*Sigma-Aldrich,* USA) and 9.9 µl lactic acid (90%; *Sigma-Aldrich, USA*) were added to 1990 µl of distilled water. The pH was titrated to a value of eight by adding sodium hydroxide solution (NaOH, 5 M). In the end, 3.5 mg nicotinamide adenine dinucleotide (98%; *Roche, Germany)* and 6 mg nitrotetrazolium blue chloride (≥ 90%; *Sigma-Alrich, USA)* were added. Samples were defrosted for ten minutes and incubated with the LDH-stain solution for 2.5 h at 37°C in light-absence. Afterwards, slides were rinsed in 50°C clear tap water and PBS followed by 4% phosphate buffered formaldehyde fixation (*Liquid Production GmbH, Germany*). DAPI (4′,6-diamidino-2-phenylindole, *Molecular Probes, USA*) intercalating nuclear stain was applied for twelve minutes as nuclei counterstain. Samples were consecutively scanned and semi-quantitative digital image analyses were realized with Fiji using the colour deconvolution plugin and user threshold values (*Fiji ImageJ Software 1.8*). AV sample surface was measured, absolute LDH-stain positive area was assessed and staining intensity measured with *Fiji* by establishing an intensity threshold based on native AV average staining. Nuclear count was determined after fluorescent DAPI counterstain.

### ECM analysis

Parallel sections (5 µm) of pAV specimens analysed for LDH activity were stained to implement a detailed ECM remodelling analysis. The stained area was semi-quantitatively assessed with *Fiji* and relativized against tissue cross-section area by using the colour deconvolution plugin and user threshold values.

MOVAT pentachrome stain demonstrates a thorough picture of ECM components and composition [35, 41, 59, 71]. Pentachrome stain was quantified with *Fiji* to calculate glycosaminoglycans and collagen fibres. Picrosirius red was used to investigate collagen fibre abundance [4, 21, 23, 72, 73]**.** The positive stained area was calculated with *Fiji*. Alizarin red staining allows investigation of AV tissue calcification [15, 19, 35, 41, 58, 74]. All histological stainings were performed according to standard protocols.

Immunohistochemistry (IHC) staining procedures were used to investigate physiological and/ or pathological VEC and VIC differentiation. The *A-2547* mouse monoclonal α-smooth muscle actin (α-SMA) antibody was applied *(Sigma-Aldrich, USA; Dilution: 1:50000)* [71]. Cluster of differentiation 31 (CD31) is expressed on the surface of endothelial cells. VEC proliferation and migration in tissue culture systems can be monitored. The mouse monoclonal *MCA 1746GA* CD31 antibody was used* (BioRad, USA; Dilution: 1:250)* [71]. Endogenous peroxidases were blocked with 1% hydrogen-peroxide solution and free epitopes kept unbound with 2.5% horse serum (Vector laboratories, USA). Retrieval buffers were not required because cryosections were used. The anti-mouse immunoglobulin kit was used as secondary antibody system (*MP-7402 Vector laboratorie*s, USA). Positive stained AV areas were quantified with *Fiji*.

### Statistical assessment

Experiments were performed with pAV (*n* = 6) and with hAV (*n* = 3) tissues. The number of analysed samples is mentioned in the results section but at least three individual AV tissue specimens were assessed. Resulting data were stated as mean ± standard deviation. Hypothesis testing for the assessment of statistical significance was computed by variance analyses with one- or two-way ANOVA and post-hoc analyses with *Tukey* multiple comparison testing using the software *PRISM* (*Graphpad Software, Inc., USA*). Null hypotheses (H_0_) were rejected if *p* < 0.05.

## Results

### MPS—TIC tissue culture

The TIC in conjunction with the pump chip allowed operation within a common cell culture incubator controlling humidity, temperature and pH. Due to modular setup and periodic *static* viability assessment, both MPS–TIC system and cell culture wells could be disinfected or replaced every second day. Suture-based *non-touch* tissue fixation within the TPU ring and insertion in the TIC proved successful and sterility was maintained for up to 26 days until scheduled setup termination.

### Particle image velocimetry

**Fig. 5:**
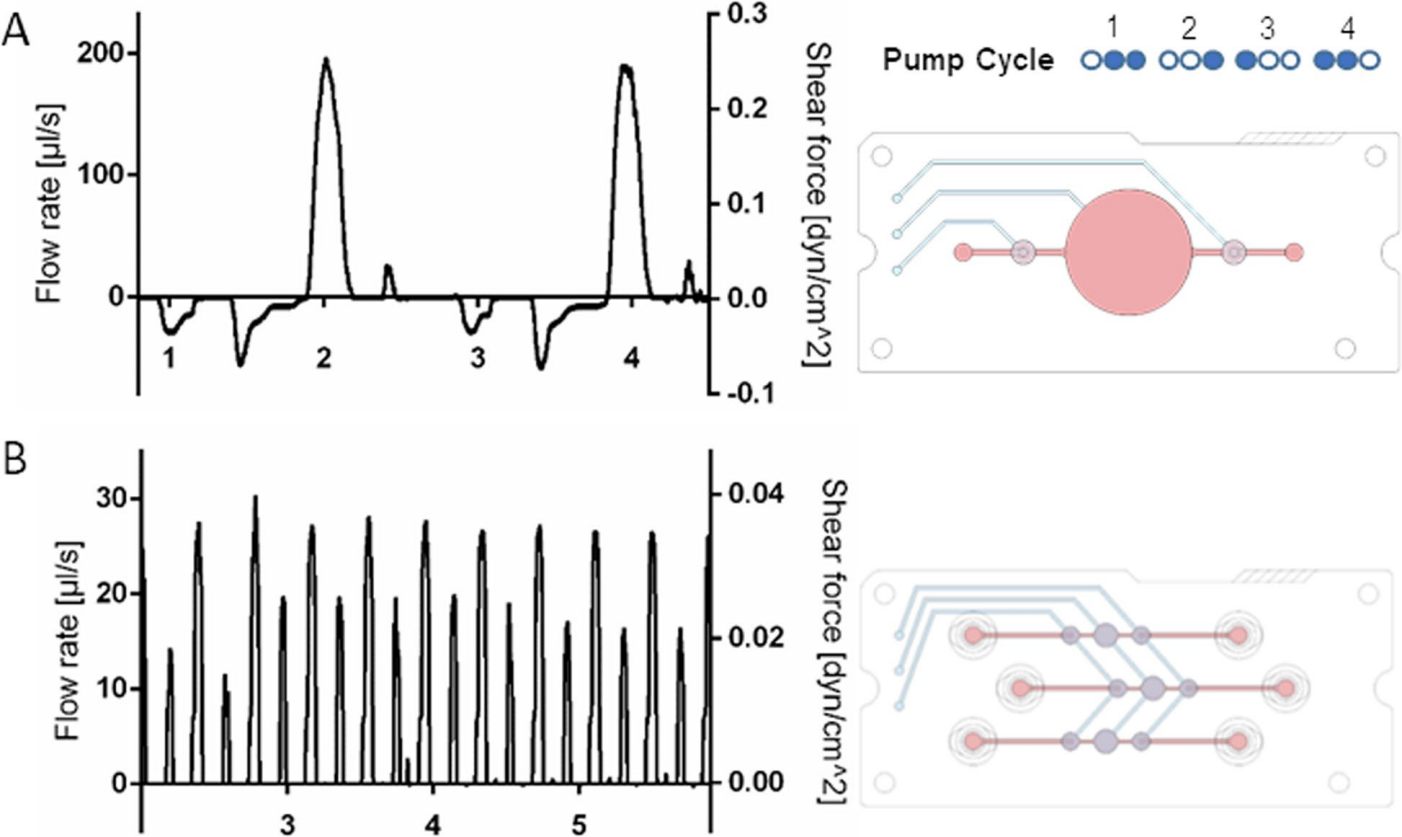
Pulsatile *dynamic* culture medium propulsion: The pneumatic pump cycle is illustrated and the flow rates in high flow setup (**A**) and low flow setup (**B**) were measured by particle image velocimetry (PIV). Cycle-specific shear forces were computed (filled circle: pneumatic pressure applied, empty circle: vacuum applied; Abscissa: seconds)

The larger pump chip that was used for *high flow dynamic* setup was measured by particle image velocimetry (PIV) revealing an elevated average flow rate of 77.4 µl/s and shear forces of 0.1 dyn/cm^2^. The anterograde peak flow rate of 195.8 µl/s caused shear forces of 0.26 dyn/cm^2^ that are 6.5 times higher compared to* low flow dynamic* setup peak shear forces. Retrograde peak flow rates of 56 µl/s caused shear forces of 0.07 dyn/cm^2^ and an oscillatory flow character (Fig. 5A)*.* The *low flow dynamic* setup provided an average culture medium flow rate of 13.4 µl/s and shear forces of 0.017 dyn/cm^2^. Simultaneously, peak flow rates of 30.4 µl/s and shear forces of 0.04 dyn/cm^2^ were computed (Fig. 5B).

### Bimodal viability assay

**Fig. 6:**
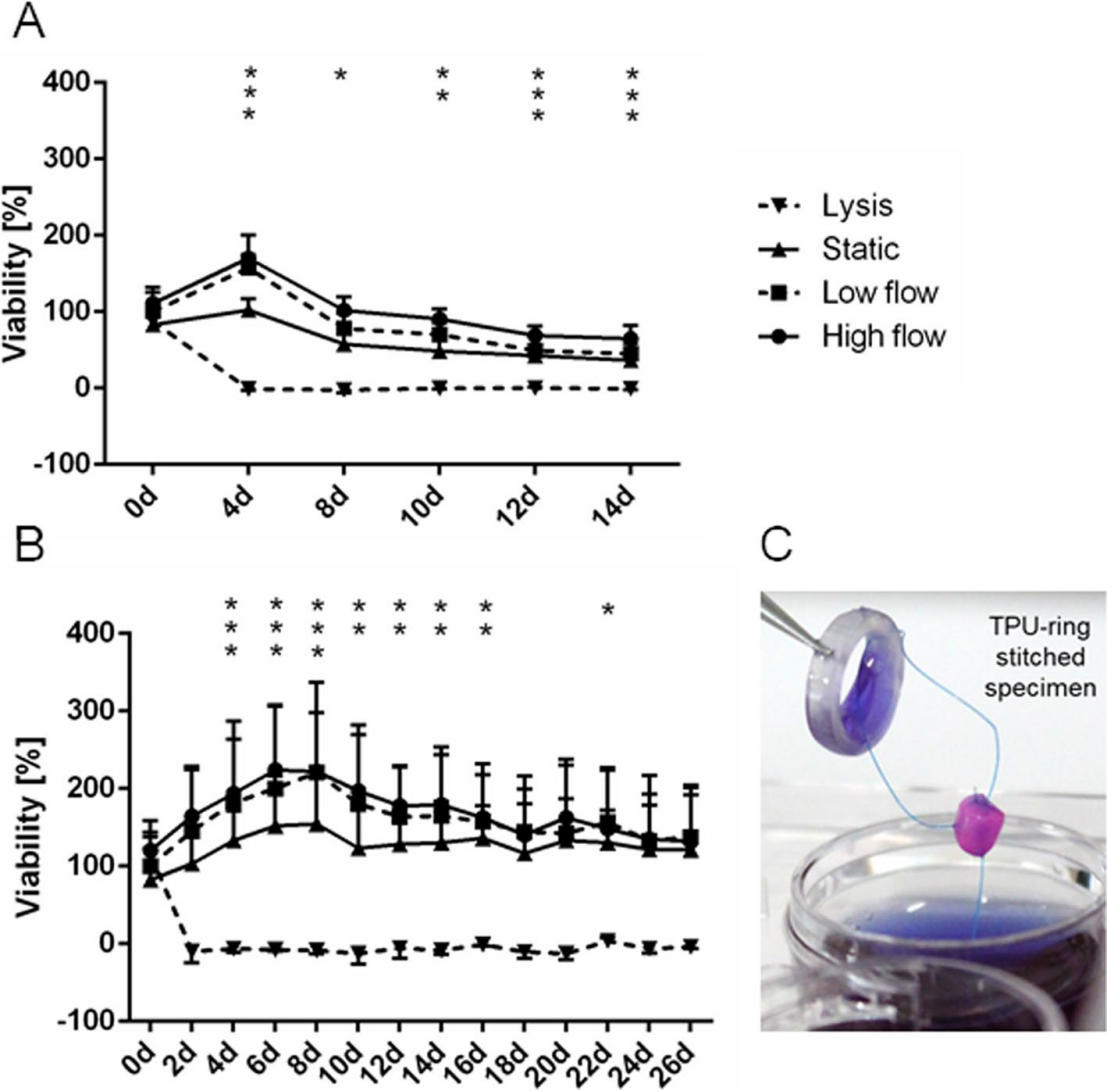
Resazurin reduction viability assessment in *dynamic* and *static* AV tissue culture: **A** pAV tissue specimens were incubated for 14 days. Non-invasive resazurin reduction viability assessment was performed on days 0, 4, 8, 10, 12 and 14. (*n* = 6); **B** hAV tissue sections were incubated for 26 days. Resazurin reduction assays were conducted every second day. (*n* = 3); **C** AV from *dynamic* setup stitched to the TPU ring after 2 h of *static* viability assessment (two-way ANOVA; * *p* < 0.05, vertical asterisks for respective setup)

For long-term viability assessment of pAV tissue specimens, the RR assay was applied immediately after excision and after 4, 8, 10, 12 and 14 days in the respective setup (Fig. 6A). Initial metabolic activity was comparable due to prior mass scaling, even though certain tissue heterogeneity was observed. After 4 days of incubation the *high* and *low flow dynamic* setups revealed a significantly increased viability of 170 ± 30.78% and 157.2 ± 17.27% (Fig. 6A) while *statically* incubated AV tissue sections exhibit an insignificant increase in these initial reduction rates to 102.3 ± 14.71%. Significant viability reduction was observed after 8 days in *statically* incubated AVs with a viability of 57.67 ± 23.03%. After 10 days, significant viability reduction was assessed in *low flow dynamic* setup with 70.33 ± 15.36%. After 12 days of *high flow dynamically* incubation, a significantly reduced viability of 69.17 ± 12.24% was noted. At the end of the experiment after 14 days, the *low flow dynamic* setup conserved a viability of 45.17 ± 13.88% whereas *static* comparison showed a viability of 36.33 ± 9.97%, revealing no significant difference between both conditions. In contrast, the *high flow dynamic* setup in conjunction with the large pump chip showed a significantly higher viability of 64.67 ± 17.21% after 14 days. No resazurin conversion was observed in the *lysis* control samples after Triton X-100 (0.25%) treatment.

Tissue segments of hAVs were equally assigned *high flow*, l*ow flow dynamic* setups, *static* and *lysis* controls. The resazurin reduction assay was conducted every second day for 26 days. Initial reduction rate indicated comparability and in succession, a viability gain was notable (Fig. 6B). On day 14, *high flow dynamic* setup remained at an elevated reduction rate of 179.0 ± 74.59%, *low flow dynamically* incubated hAV tissue sections at 164.7 ± 79.03% and *static* controls at 130.3 ± 53.3%. No significant viability difference between *dynamic* and *static* setups was noted. RR assay results interpretation of hAV tissue sections remain challenging because the overall resazurin reduction rate is approximately 2.5 times lower compared to pAV tissue specimens. An effect that is caused by significantly higher number of viable VICs in pAV tissue [50]. *Lysis* controls did not reveal any reduction rate.

**Fig. 7:**
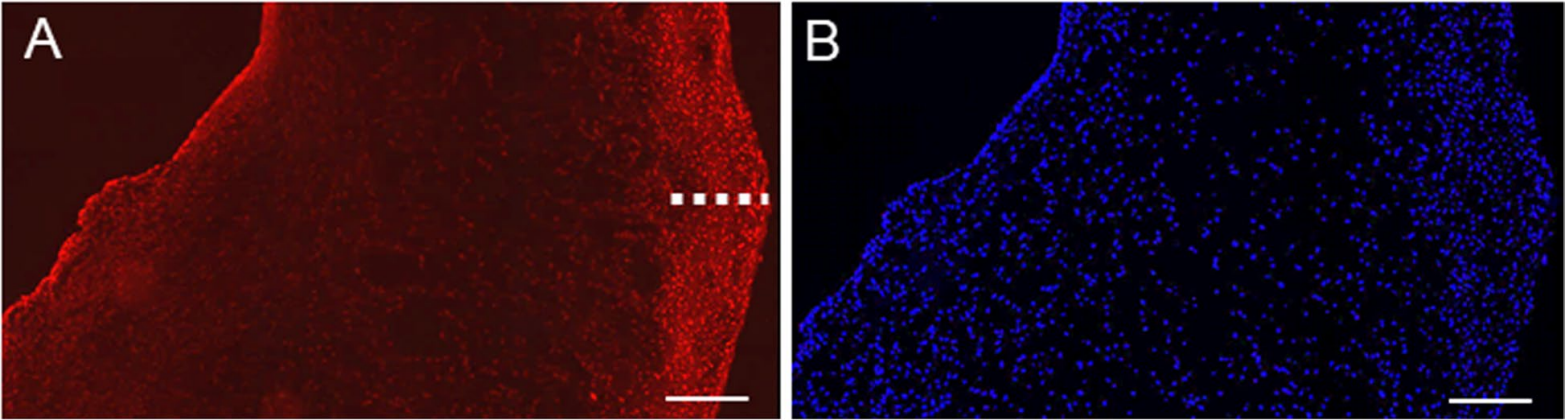
Visualization of native pAV tissue resazurin penetration: The dashed indicator bar displays peak penetration depth with high resorufin concentration. **A** The fluorescing resazurin reduction product resorufin is visible in red. **B** The DAPI-stained nuclei impose blue-fluorescence (*n* = 3, representative samples shown, Scale bar: 200 µm)

Efficacy of the resazurin reduction assay depends on effective substrate penetration. The investigation via fluorescence microscopy stated a peak resazurin concentration diffusion depth of 174.7 ± 43.87 µm (Fig. 7). Reduced resazurin reduction in the consequence may monitor merely superficial cellular enzymatic activity that can depend on layer ECM density and individual tissue subsection.

**Fig. 8:**
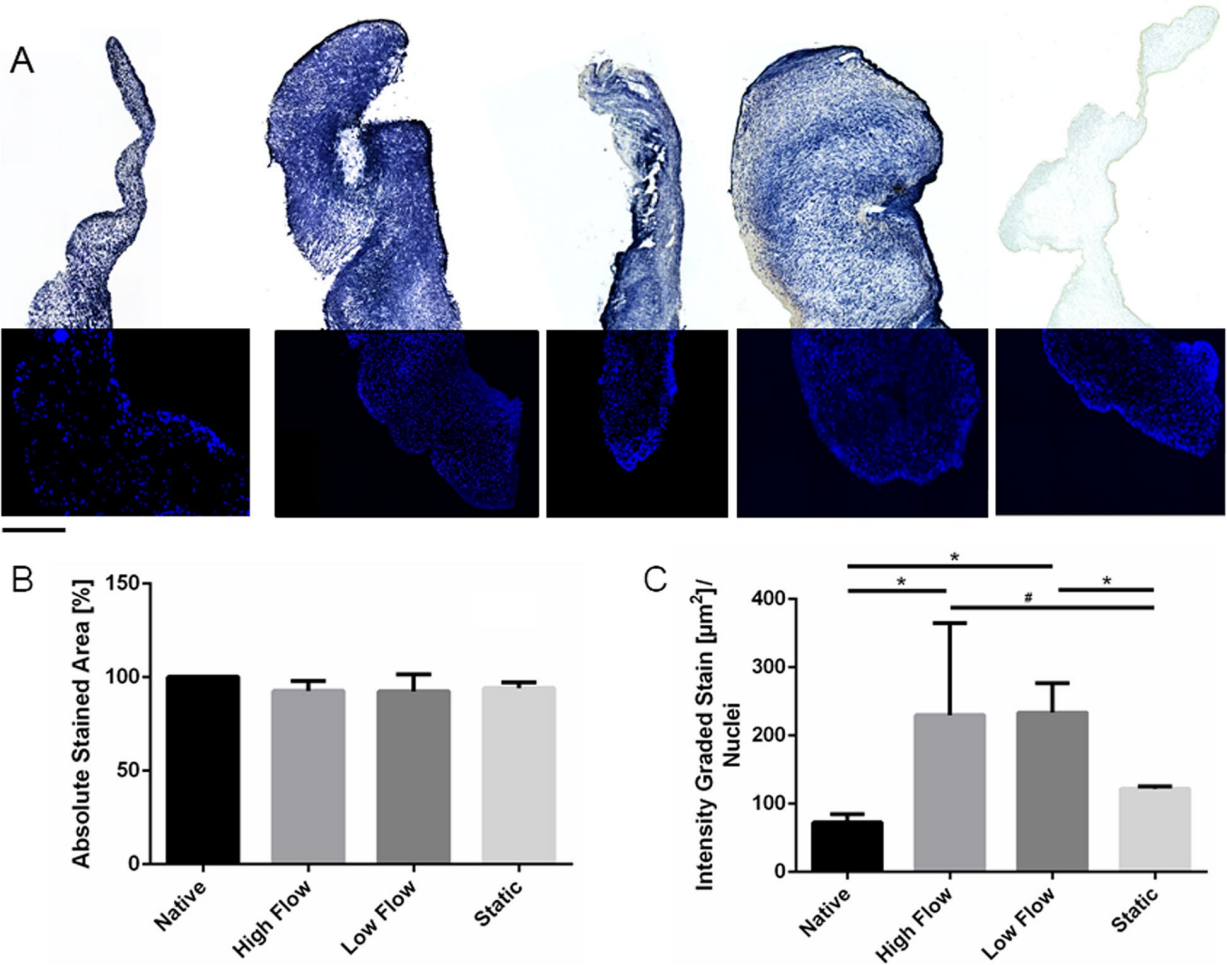
End-point viability assessment by LDH cryosection stain after pulsatile *dynamic* vs. static AV tissue culture: **A** LDH-viability stain performed on pAV tissues at the beginning of the experiment and following 14 days of incubation under *high flow dynamic*, *low flow dynamic*, *static* and death tissue conditions. (Left to right, representative samples shown, *n* = 6, scale bar: 500 µm); DAPI nuclear counterstain is visualized in the lower part of the tissue section; **B** Positively stained areas were quantified and relativized against cross section surface; **C** Staining intensity variation was metrically assessed by applying a colour threshold (two-way-ANOVA; * *p* < 0.05; # *p* < 0.1)

The LDH viability stain was conducted in parallel with the RR assay. Cellular demise as shown by the RR assay with viability loss of more than 50% was not supported. Respective findings support the assumption of predominantly superficial cell death. On the one hand, tissue viability was inferred from the absolutely stained cross section area (Fig. 8B)*.* No significant reduction was observed and the *lysis* control did not depict any stained areas. On the other hand, tissue stain intensity was quantified and tissue shrinkage was normalized by relativizing the measured LDH-viability stain area against nuclei in the cross section. The result demonstrates the individual cellular potential of substrate turnover, which can be interpreted as metabolic activity. The metabolic activity increased significantly following *high* and *low flow* dynamic incubation to 229.8 ± 135 and 233.6 ± 43.2 µm2 intensely positive stained area per nucleus compared to values in native tissues of 72.45 ± 11.95. The *static* control showed 121.9 ± 3.41 µm^2^ intensely positive stained area per nucleus (Fig. 8C). No significant increase was observed in *statically* incubated AVs compared to native control.

### Histological analyses

**Fig. 9:**
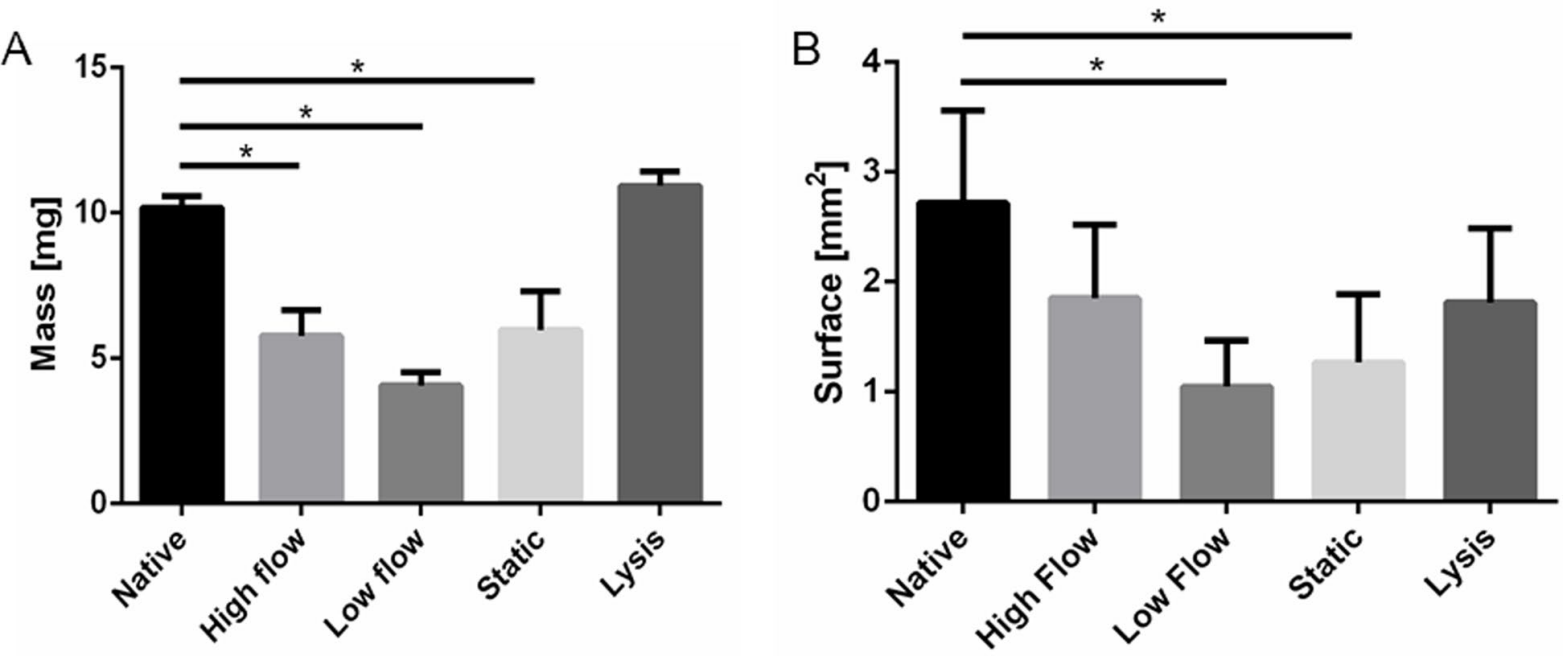
Mass reduction and tissue shrinkage in AV tissue culture settings: **A** pAV tissue mass was measured at the beginning of the experiment and after 14 days of incubation; **B** Cross section areas before and after the experiment (14 days) were opposed (*n* = 6; two-way-ANOVA; * *p* < 0.05; no significance shown for lysis control)

After 14 days of pAV tissue section incubation, a significant mass loss was detected for tissue samples in each setup except *lysis* controls. Initial tissue sections were mass-normalized to 10 ± 0.5 mg. pAV subsections after 14 days of *high flow dynamic* incubation exhibited a mass of 5.77 ± 0.87 mg and *low flow dynamically* incubated tissue of 4.07 ± 0.46 mg. AVs from *static* comparison depicted a mass of 5.97 ± 1.33 mg and *lysis* control gained mass to 10.93 ± 0.5 mg (Fig. 9A). The AV cross section area decreased significantly in *low flow dynamically* and *statically* incubated samples from 2.72 ± 0.84 mm^2^ to 1.05 ± 0.42 and 1.27 ± 0.61 mm^2^ respectively. Samples under *high flow dynamic* incubation showed no significant alteration with a surface of 1.85 ± 0.67 mm^2^ (Fig. 9B).

**Fig. 10:**
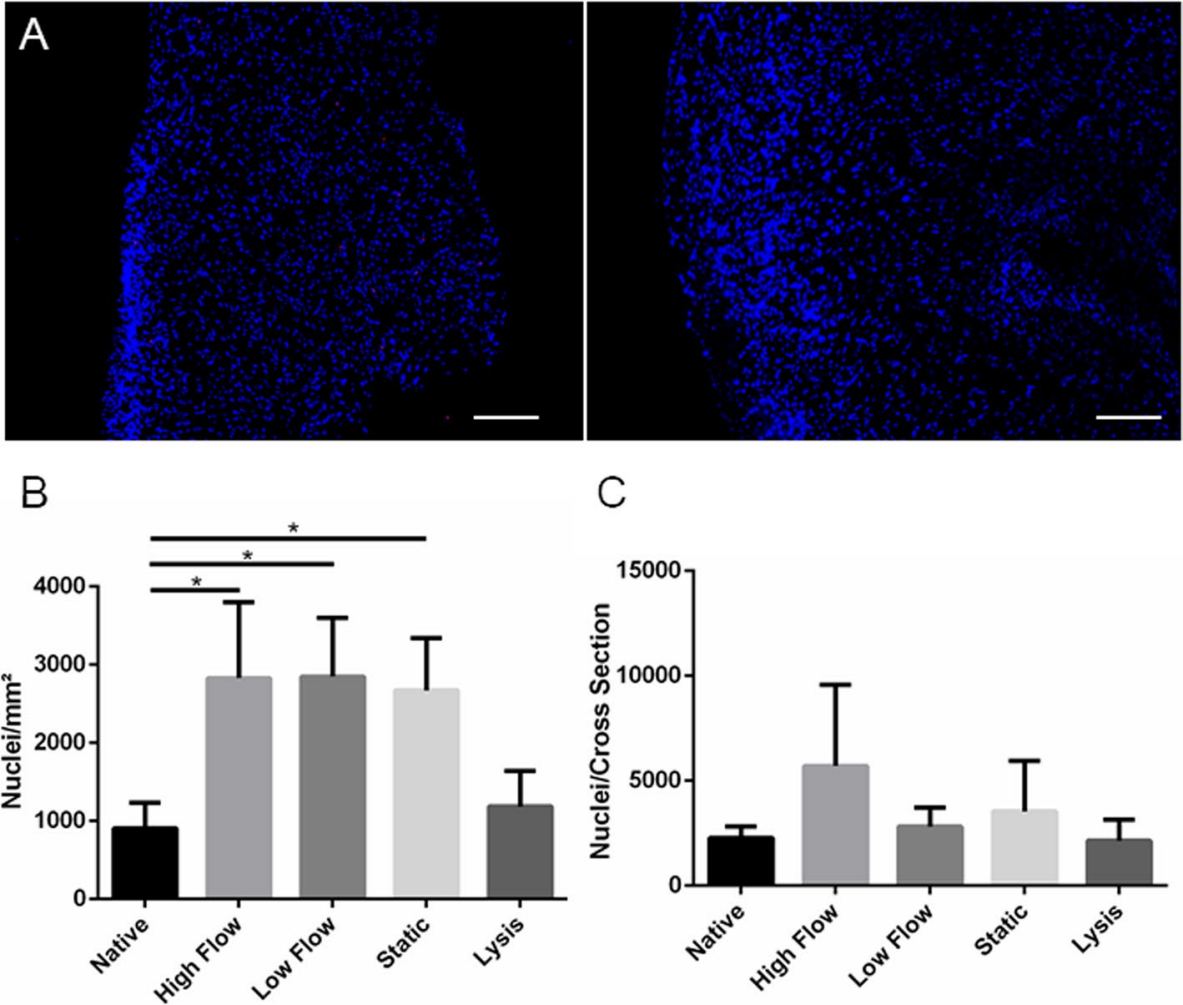
Cellular density after pulsatile *dynamic* vs. *static* AV tissue culture: **A** Density alteration is visible in DAPI stained nuclei of native (0 days, left) and statically incubated pAV tissue Sects. (14 days, right). **B** Cellular density was assessed and relativized against AV cross section area due to valvular shrinkage (*n* = 6, Scale bar: 300 µm; two-way-ANOVA; * *p* < 0.05; no significance shown for lysis control)

Quantification of nuclei after DAPI staining was realized to investigate nuclear density. All cultured tissues exhibited a significant increase of nuclear count per section area after 14 days of incubation compared to native pAVs. Comparable nuclear density was observed in *lysis* control. Native pAV tissue specimens depicted 908.3 ± 324.6 nuclei/mm^2^, *high* and *low flow dynamically* incubated pAV tissue subsections 2828 ± 971 and 2848 ± 754.9 nuclei/mm^2^ respectively and *static* controls 2673 ± 669.4 nuclei/mm^2^ (Fig. 10B). Relativizing the increased nuclear density against reduced AV cross-section area resulted by contrast in non-significant elevation of nuclear count (Fig. 10C).

**Fig. 11:**
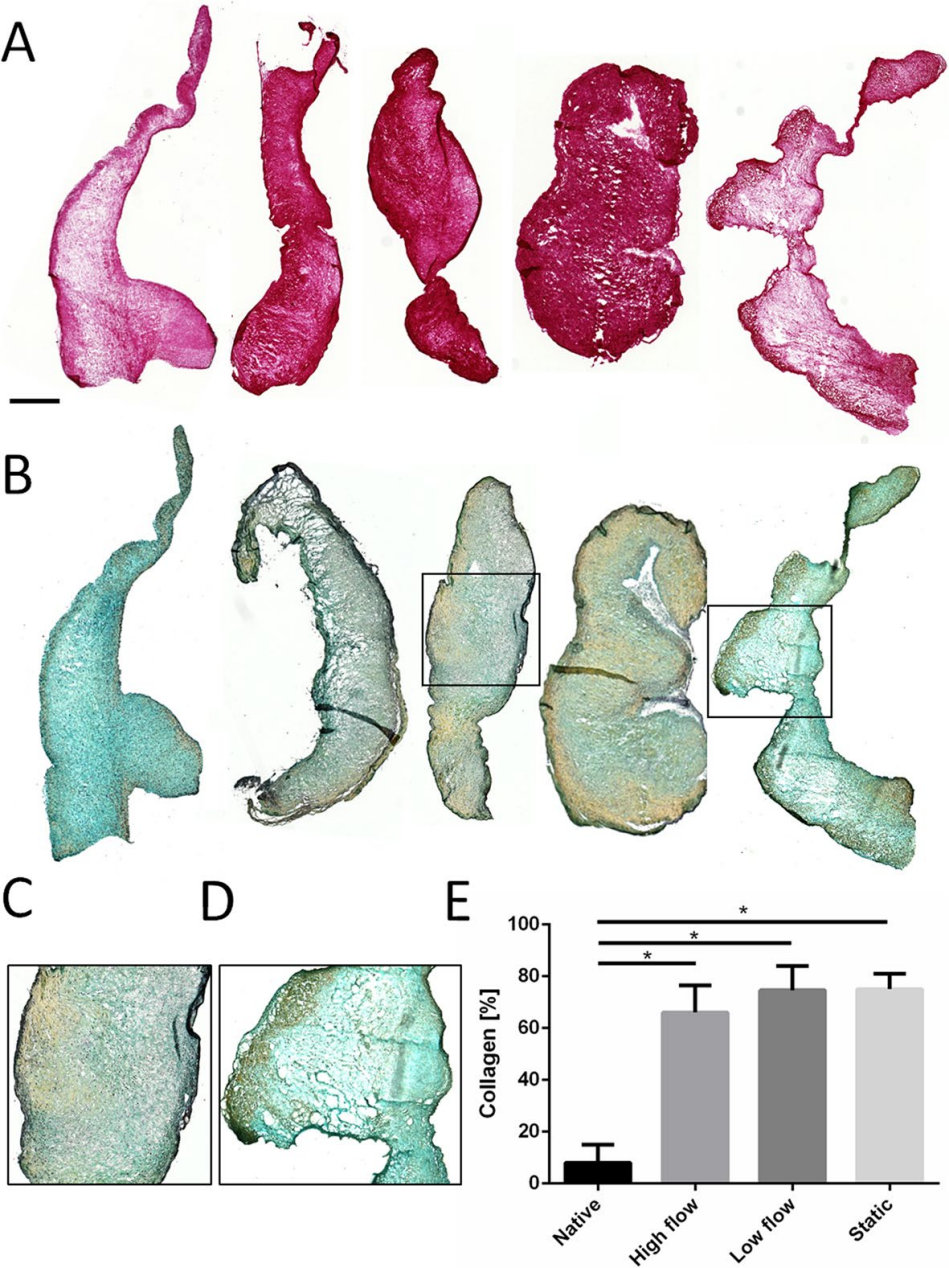
Collagen quantification and tissue laminae after *pulsatile dynamic* vs. *static* AV tissue culture: **A** pAV tissues were stained with picrosirius red at the beginning of the experiment and after 14 days of *high* and *low flow dynamic*, *static* and *lysis* incubation conditions to reveal collagen fibres (left to right, representative samples shown, *n *= 3, scale bar: 500 µm); **B** MOVAT pentachrome stain was applied to assess trilaminar tissue stratification (**C**). A sponge-like ECM configuration appeared in lysis control (**D**); **E** Collagen fibres were quantified according to picrosirius red stain and relativized against cross section surface (two-way-ANOVA; * *p* < 0.05)

Staining pAV tissue subsections with picrosirius red reveal a higher abundance of collagen fibres at the level of the fibrosa in native AV whereas only sparse collagen is found in the centre of the AV and at the ventricularis layer. After 14 days of incubation, *dynamically* and *statically* incubated pAV tissue samples showed a significant increase of collagen fibres by 71.97 ± 6.14% positive cross section staining compared to native AV specimens that exhibited 8.07 ± 7.04% (Fig. 11A, E). Cryo-fixed pAV tissue samples were stained with MOVAT pentachrome for detailed ECM analyses. The native pAV tissue section ventricularis layer contained abundant elastin fibres, the spongiosa glycosaminoglycans and the fibrosa collagen fibres. The stain revealed a maintained AV tissue stratification in *dynamic* and *static* setups after 14 days of incubation (Fig. 11B, C). Quantifying the yellow and the blue dye that represent collagen fibres and glycosaminoglycans respectively, showed no significant difference between the *dynamic*, *static* setup and native AVs (data not shown). Sporadically and non-systematically sponge-like ECM morphology of pAV tissue specimens were detected e.g. in the lysis control group (Fig. 11D). The alizarin red stain did not depict any calcification in the native control or in the setups after 14 days of incubation (Supplementary Fig. 1).

**Fig. 12:**
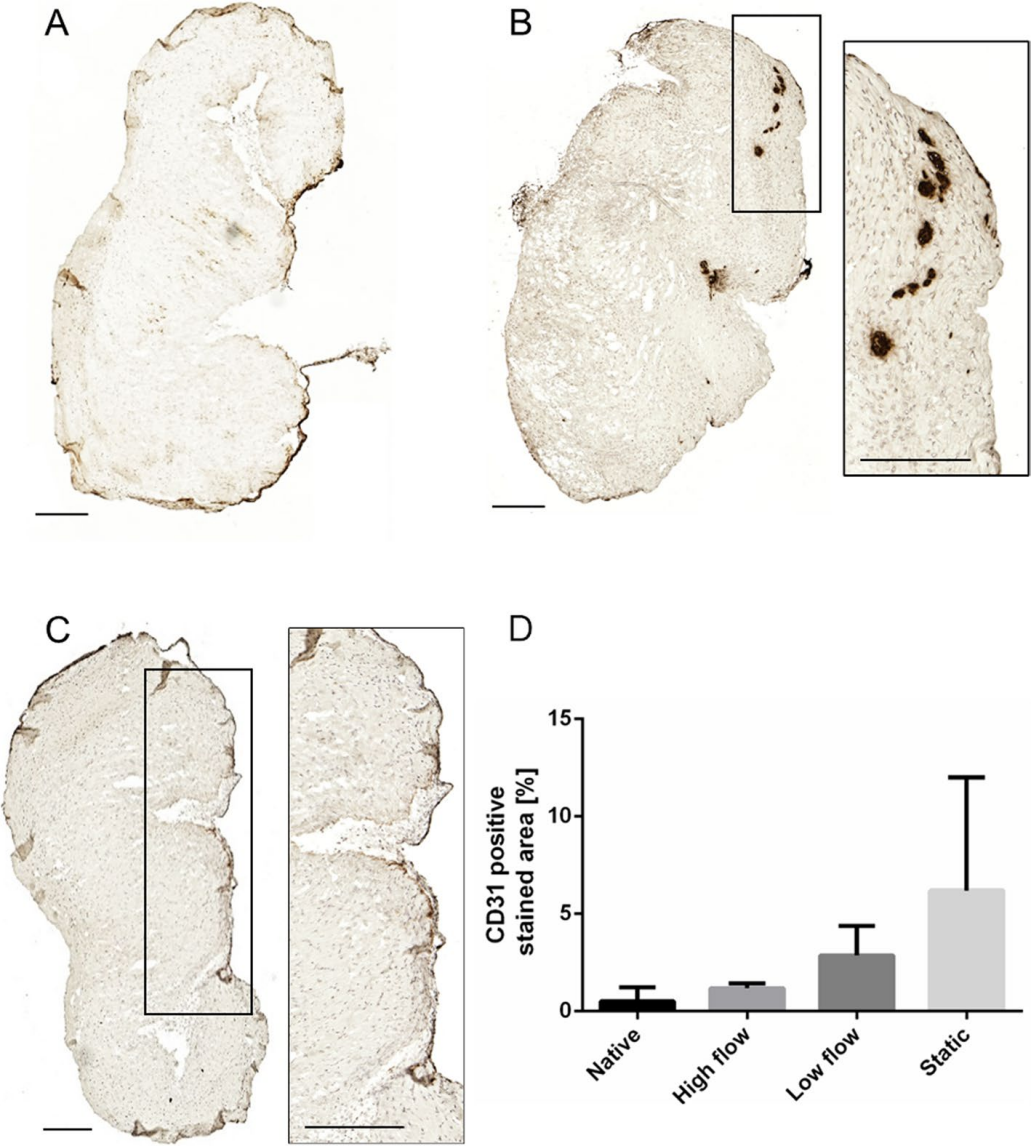
α-SMA and CD31 immunohistochemistry staining of *dynamic* vs. *statically* cultured AV tissue sections: **A** *Statically* incubated pAV specimen after 14 days is shown (left, *n* = 3, representative sample shown); **B** Preliminary AV incubation setups under static conditions demonstrated increased focal α-SMA expression after 21 days; **C** CD31 epitopes of pAV tissue specimens are stained after 14 days of *static* incubation. The magnification shows neo-endothelial lining; **D** Quantification of CD31 expression (*n* = 3, scale bar: 200 µm; two-way-ANOVA; * *p* < 0.05)

Immunohistochemistry staining of α-SMA revealed no significant difference between 14 days incubated setups and native control (Fig. 12A*; *Supplementary Fig. 2). Preliminary experiments showed focal upregulations of α-SMA epitopes after 21 days of incubation under *static* conditions (Fig. 12B). The quantification of CD31 revealed an insignificant upregulation to 6.19 ± 5.8% positive stained cross-section area in *statically* incubated AV specimens (Fig. 12D). Endothelial lining persisted over the incubation period of 14 days. Certain specimens, especially *statically* incubated, delineated endothelial linings spanning over tissue folds (Fig. 12C).

## Discussion

The calcific aortic valve disease (CAVD) resides among the world’s most prevalent culprits of cardiovascular disorders [1, 2, 75]. There is an implicit need of profound investigation, especially because of lacking conservative treatment options [1, 45]. Thorough understanding of the multifactorial pathophysiological processes underlying CAVD may provide novel prophylactic strategies for people at risk as well as curative treatment options for patients already suffering CAVD.

AVs are exposed to systolic blood pressures of up to 150 mmHg in hypertonic patients and blood flow volumes of 5 to 15 L per minute for a patient’s lifetime without any interruption [76, 77]. Due to the complex and extreme microenvironment of heart valves, it remains challenging to introduce a physiological in vitro model system reflecting these conditions. Application of 2D-cell cultures allow basic experimental setups [9, 21–23, 26] but there are non-representative model attributes such as physiological cell–cell and cell–matrix interaction as well as biomechanical and -chemical microenvironmental influences [6, 25, 41, 43, 58, 78]. Limitation of calcification in contrary is possible by adding pro-calcifying or osteogenic media [79–81]. More realistic representation was achieved using animal models but altered microanatomy and absent/reduced calcification potential limited model applicability [9, 15, 26–29, 31, 32, 45]. Pro-calcifying or osteogenic substance-based induction is not possible in animals but spontaneous AV calcification could be at most observed in porcine samples, accelerated by supplying high cholesterol and fat diet [28, 82–84]. Wild-type mice and rabbits in parallel do not exhibit spontaneous CAVD lesions [28].

MPSs have been introduced using 2D-cell cultures as well as 3D-tissue cultures. These bioreactors allow long-time incubation under dynamic conditions and simulation of biomechanical properties such as specific shear forces, pressure, mechanical drag together with biochemical replication of tissue hypoxia and application of pro-calcifying media (Table 1) [3, 4, 16, 34–41, 48, 49, 78, 85–87]. Because of the immense diversity in AV tissue implementation und numerous adaptations of certain physiological parameters, MPS became a major bridging element between 2D-cell cultures and animal models with the aim to better reflect the human in vivo situation and reduce animal experiments to a reasonable amount [34]. Nevertheless, there is a persisting challenge regarding result convergence with human pathophysiology [30]. Still the application of MPS is limited in regulation of shear forces, pressure or the simulation of clinical factors such as chronic kidney disease. By selective adaption of medium condition the MPS models can be further improved and the dynamic incubation in human blood is envisioned. In addition, until now, there are no MPSs systematically applying hAVs or tissue segments. Due to limited access of the material this can be advantageously achieved in the presented intermediate MPS-setup.

### Novel MPS-TIC system

The presented study describes a novel MPS for intermediate sized AV tissue incubation of both porcine and human origin under pulsatile dynamic conditions coupling the pneumatic pump system to tissue incubation chamber. *Static* incubation inheres model disadvantages because biomechanical simulation of extreme AV microenvironment is not reflected [39, 41, 58]. AV tissue segments of intermediate size were applied that allow yielding of four to five samples per leaflet in parallel. In consequence, technical replication is granted as well as improved comparability under various setup regimes. Beside higher material costs, larger scaled setups that apply entire AV roots require evaluation in respect of inter-individual differences between valve donors [35]. Feasible incubation periods of 14 – 26 days already pose the potential for reliable CAVD pathogenesis and investigation of substance intervention. Given the fact that CAVD develops over decades, it is presumable that short-term in vivo and in vitro model systems still are critically limited and MPS culture periods of 14 to 26 days should be extended best possible. Furthermore, AV remodelling depends on biomechanical influences, also referred to as mechanotransduction. The impact was proven by various in vivo and ex vivo setups [4, 25, 35, 38, 57]. Continuous, non-pulsatile flow for example leads to increased aortic wall thickness, smooth muscle content and collagen quantity in human aortae. The elastin content reduces and results in increased vessel stiffness [88]. The novel MPS proved applicable for tissue incubation under *dynamic* pulsatile conditions. The *low flow dynamic* setup achieved peak shear forces of 0.04 dyn/cm^2^ [33]. In contrary, there are physiological shear forces of approximately 0 to 79 dyn/cm2 acting on the lamina ventricularis and -8 to 10 dyn/cm2 at the level of the lamina fibrosa in hAVs [4, 37, 40, 42, 56, 89, 90]. To realize physiological shear forces, a larger pump chip was developed and applied in *high flow dynamic* setup. It allowed peak shear forces of 0.26 dyn/cm2, still not reflecting in vivo conditions but already realizing a 6.5 folds increase. Since the larger pump chip evacuated culture medium volume critically for system integrity, an attenuation element was implemented also allowing airless MPS culture by acting as a bubble trap.

### Bimodal viability assessment

Reliable viability assessment is required to avert bias or data misinterpretation caused by tissue proliferation or mortification. The bimodal approach of introducing both a non-invasive resazurin reduction assay and a histological LDH-based viability stain complements current viability estimations based on apoptotic fragmentation [37, 56], TUNEL assessment [3, 4, 37] or LDH membrane integrity assay [41]. Latter was applied by *Weber *et al*.* but failed applicability in AV tissue segments, due to insufficient assay sensitivity of LDH solute and solvent ratio [33, 50]. Resazurin reduction has been applied in 2D-, 3D- cell and tissue cultures [68, 69, 91–93]. Substrate diffusion limitations [44, 69] led to a thorough evaluation and protocol adaptation [50]*.*

The resazurin reduction assay enabled a non-invasive instrument of AV tissue viability assessment and demonstrated an advantageous viability in *high flow dynamic* setup. Limitation of insufficient resazurin penetration depth may depend on AV layer distribution and therewith varying cellular density that in turn can depend on the heterogeneity of individual AV subsection excised from leaflet coaptation region. *Sonnaert *et al. computed a resazurin diffusion depth of 700 µm, but artificial scaffolds (*Ti6Al4V)* were used instead of AV tissue [69]. *Maeda *et al. in parallel emphasised the importance of AV coaptation and associated tissue stretch for diffusion potential [44]. Passive oxygen diffusion range of unloaded AVs, for example, reaches only a depth of 100 µm at a valvular thickness of 300 – 700 µm [94, 95]. In situ AV coaptation leads to surface increases up to 20 – 30%, reducing thickness and facilitating diffusion [96]. AVs exist on the verge of hypoxia under physiological conditions and any alteration such as fibrotic thickening may hamper the sensitive equilibrium [44, 97]. The missing coaptation and reduced stretch conditions in the presented setup also may be the reason for limitations in resazurin diffusion. In consequence, tissue fixation perpendicular to the culture medium flow is projected to allow valvular coaptation and stretch forgery. The peaking reduction rate on day four in *dynamically* incubated AVs tissue setups may result from increased cell stress but remains to be elucidated in ongoing experiments. It can also be a result of increased endothelial cell metabolism due to culture conditions. Beside assay limitations, resazurin assay offers a non-invasive possibility to monitor metabolic activity and surveillance of AV tissue cell viability especially necessary for long-term culture.

Incubation of hAV tissue subsections for 26 days proved equally applicable, sterility was maintained and tissue survival demonstrated by absolute resazurin reduction. For the first time in general, it was possible to realize hAV tissue incubation within a MPS under biomechanical conditioning. Nonetheless, the viability gain throughout the experiment and especially within the first two weeks requires additional investigation. Aggravated diffusion potential in diseased AVs may have led to VEC and VIC recovery under ex vivo conditions.

In addition, tissue culture samples are removed from the dynamically incubation during RR-assay and parallel MPS-TIC disinfection. This interruption can lead to reduction of viability. After intense characterization of MPS-TIC culture process presented frequent repetition of RR-assay will be reduced or omitted for ongoing investigations.

The LDH-viability stain was implemented to investigate profound AV tissue viability in an end point analysis also allowing estimation of cellular density and ECM analyses of parallel cryosections. Quantification of stained cross sections lacked larger non-viable tissue areas and resulted in a maximum viability reduction of less than 10%. A significant increase of staining intensity and cellular density were noted in *high* and *low flow dynamic* setups as well as in *statically* incubated AVs. Concomitant tissue mass and cross-section area in these samples decreased significantly. Relativizing increased cellular density with reduced AV cross-section area revealed no significantly altered cell number compared to native tissue. Hence, excessive cellular proliferation is disproved and ECM remodelling of contraction and reduction over 14 days of incubation can be assumed. Relating staining intensity with cell number delineates significantly increased dye intensity in *dynamic* setups. Therefore, an elevated metabolic rate in *dynamically* incubated AVs and abstained significant cell death are suggested. The initial resazurin-based viability estimation could therefore not be confirmed by LDH-viability stain and may be explained by insufficient substrate penetration conjoined with superficial cellular decay. These results emphasise the importance of multimodal viability assessment in upcoming ex vivo MPS applications. Otherwise, pivotal bias may be inherent to future AV tissue investigation, also because AV cell death, especially apoptotic VICs, were described to be responsible for calcification-core constitution in CAVD [9, 98–100].

Former observations of broad central tissue mortification in *static* controls proved non-reproducible [33]. Most likely, unfavourable AV tissue contraction and subsequent diffusion deterioration were responsible for the observation (*n* = 6). Also culture dependent limitation of oxygen diffusion in AV tissue statically incubated with covering medium or oxygen consumption are relevant [101] and can depend on individual specimen. Choosing non comparable locations from heterogeneous AV architecture for sampling may constitute another explanation [31]. The phenomenon may occur again and must be considered in respect of result interpretation. The analysis of entire tissue culture specimen by staining every histological section of the sample is envisioned to define the impact of cutting depth and realize an impression of LDH viability in the 3D tissue structures.

### ECM analyses

The elucidation of CAVD pathophysiology requires a reliable *ex vivo* model system. Because biomechanical and -chemical equilibrium changes after AV explantation, it is mandatory to evaluate morphological AV transitions. In the presented study, trilaminar ECM structure maintenance was proven by MOVAT pentachrome stain which coincides with published findings [4, 12, 35, 39, 41, 59]. Occasional alteration of the ECM structure may be explained by varying collagen fibre architecture in each individual AV and the respective tissue subsection [31]. pAV tissue specimens experienced shrinking and mass decrease in *dynamic* and *static* culture. *Zabirnyk *et al. described similar observations and consequently applied antimyofibroblastic medium to prevent AV contraction [39]. The success of this intervention suggests myofibroblastic VICs to be responsible for contraction. The observed mass decrease in MPS-TIC culture in parallel suggests limited synthetic activity of VICs. *Thayer *et al. [59] distinguished varying differentiation patterns of VICs in pathogenesis. The *fibroblast-like* phenotype is responsible for ECM maintenance, the *myofibroblast-like* differentiation enables contractility and ECM synthesis and *smooth-muscle-like* VICs are defined solely by contractility without matrix synthesis [15, 59, 102]. Although *smooth-muscle-like* VIC differentiation fits tissue contraction and mass reduction, subclassification is not supported by an increase of α-SMA expression. Missing induction indicates absent VIC differentiation after 14 days of incubation. Initial pAV tissue samples investigated after 21 days of *static* incubation in contrary, exhibited α-SMA-positive cells. These preliminary results have to be proven in upcoming investigations. Alternatively, reduced stretch, pressure and shear forces in the presented ex vivo MPS-TIC system are suggested to provoke tissue contraction and shrinkage realized by tissue inherent strain and already existing VICs of myofibroblast phenotype [39]. Mass reduction in parallel may be caused by an imbalance of fibrotic and proteolytic processes induced by matrix metalloproteinases (MMP) and tissue inhibitor of MMP-activity as well as TGF-β alteration [17, 19, 103, 104]. Changes in tissue water content due to incubation in the artificial culture medium and concomitant potential increase of tissue stiffness will be contextualized.

Collagen content increased significantly in both *dynamic* and *static* setups according to picrosirius red stain and tendentious in MOVAT pentachrome stain. In congruence with *Weber *et al., occasional “sponge like” matrix transformation was found after 14 days of incubation in all experimental setups [41]. The histological morphology appears akin to myxomatous AV degeneration, a process characterized by AV thickening, fibrosa disruption, ECM loosening, collagen fibre type VI fragmentation, acid mucopolysaccharide accumulation and also VIC activation to myofibroblast phenotype [105, 106]. pAV tissue section thinning, intact fibrosa, absence of α-SMA expression and preservation of collagen fibres however contradict myxomatous phenotype in the presented study. The observed ECM transformation rather exhibits early-state CAVD properties, which in contrary are characterized by collagen fibre accumulation together with elastin reduction leading to increased AV stiffness and altered hemodynamic properties [13–15, 17, 19, 43]. Since elastin fibres are described to be fragmented and reduced in CAVD pathogenesis, histological evaluation of *dynamically* and *statically* incubated pAV tissue specimens is projected. Mechanotransductive regulation of glycosaminoglycans was demonstrated previously as well but abstained significant alteration in this study following *dynamic* or *static* incubation [38, 57].

Furthermore, disrupted endothelium, VIC proliferation and activation characterize CAVD pathogenesis [9–11, 13–15, 17, 43]. In the presented study, endothelium integrity was largely maintained but occasional disruption may still constitute earliest CAVD-specific lesions. IHC staining of VEC epitope CD31 trended to higher expression in the samples following *static* incubation, in which bridging of endothelial linings over tissue folds were striking. This phenomenon did not occur in *dynamically* incubated AVs and emphasises lacking tissue stretch under *static* conditions. An increased fraction of intact VECs may consecutively sustain improved VIC renewal by endothelial-to-mesenchymal transition (EndMT), VIC-VEC interaction and VEC-driven AV protection [107, 108]. AV contraction in parallel with endothelial covering may also aggravate diffusion properties and provoke central tissue mortification as observed in previous setups [33]. Preliminary datasets reveal a trend for lower mRNA-expression of TGF-β in dynamically cultured (*high flow setup*) porcine AV tissue specimen compared to static culture (data not shown). Nevertheless, in all cultured samples the value was higher than in the original native tissues. This can impact processes such as EndMT [109], which was not investigated in detail so far for the presented system. EndMT markers are regulated time dependent and there is no consensus for an exact molecular and functional definition of the mechanism [110]. Ongoing work examines expression profiles of porcine AV tissue specimen cultured in the MPS-TIC with statically cultured and native counterparts in detail also comparing markers for EndMT. In addition, MPS limitations in shear forces can lead to the induction of EndMT. Low shear stress in comparison to pathological high shear forces but also steady vs. oscillatory shear forces resulted in alteration of EndMT-marker expression [110–112].

Objective of a dynamic pulsatile AV tissue culture MPS is the maintenance of physiological AV tissue viability and ECM organization [113]. In addition, the MPS should allow disease state mimicry by directed aberrant biomechanical modulation avoiding pro-calcifying or -degenerative additives to investigate related processes. Examination of substance intervention for CAVD prophylaxis or regression are resulting challenges [113, 114]. Biomechanical parameters of dynamically incubated AV tissue as described in this study accomplished early-disease state ECM reorganization in parallel with elevated metabolic activity. Underlying cellular regulation processes are currently under investigation by mRNA expression profile analyses.

Future adaptations should include an improvement of biochmechanical simulation. Shear forces acting on the AV for example will be increased by reducing the inner diameter of the TIC cylinder or adjusting the pump chip dimensions. More physiological shear forces with simulation of the aortic (low oscillatory shear forces) vs. ventricular side (high unidirectional shear) can lead to better tissue homeostasis and keep VEC morphology and expression patterns near the native state [115]. This can further improve AV tissue viability and maintenance in the MPS-TIC culture. Improved larger pump chip for the *high flow dynamic* setup already approximated the extreme and complex AV microenvironment but further approach remains necessary. System pressure adaptation combined with a novel tissue fixation strategy perpendicular to the fluid vector can allow mimicry of cusp opening, closing and competent coaptation. Therefore, increased substrate diffusion properties essential for tissue viability should be obtained and physiological mechanotransductive requirements can be met. Hypoxia has a potential impact on AV pathogenesis following fibrotic tissue thickening in early CAVD and can be simulated using an oxygenator connectable to the MPS-TIC system. Especially the possibility of implementation and detailed assessment of hAV tissue is advantageous in the presented MPS-TIC, still with the limitation that varying age, pathology and valve integrity aggravate results interpretation.

## Conclusion

The presented study introduces the novel bioreactor MPS-TIC for incubation of AV tissue samples and a bimodal viability assessment together with an ECM analysis protocol. Particularly, hAV tissue section incubation in an MPS was demonstrated for the first time. AV tissue culture constitutes a bridging element between in vivo and in vitro experimental setups. In addition, it allows to resolve 2D cell culture model limitations such as interaction of cellular subpopulations by tissue application even of human origin. The biomechanical simulation is indispensable for investigation of CAVD (patho)physiology that is highly affected by mechanotransductive processes and is envisioned to be further adjusted.

### Supplementary Information

**Additional file 1: Supplementary Figure 1.** AV tissue calcification after pulsatile dynamic vs. static tissue culture: pAVs were stained with Alizarin red to investigate valvular calcification at the beginning of the experiment and after 14 days of high and low flow dynamic, static and lysis incubation conditions. Calcified hAV was used after explantation as positive control. (left to right, *n*=3, scale bar: 200 µm).

**Additional file 2: Supplementary Figure 2.** Expression of α-SMA and CD31 in pAV tissue after pulsatile dynamic vs. static tissue culture: pAVs (high flow dynamic, low flow dynamic, static and death tissue conditions, 14 days; left to right, representative samples shown) were stained via immunohistochemistry to verify expression of the respective marker. No significant differences were detected for α-SMA expression. Rate of CD31 positive signal was significantly higher in statically incubated samples (shown in Figure 12; scale bar: 500 µm).

## Data Availability

The datasets used and/or analyses during the current study are available from the corresponding author on reasonable request.
