# Supplementary figures and images for: Challenges of aortic valve tissue culture – maintenance of viability and extracellular matrix in the pulsatile dynamic microphysiological system
Source: J Biol Eng. 2023 Sep 28;17:60. doi: 10.1186/s13036-023-00377-1 (PMC10538250; doi:10.1186/s13036-023-00377-1)

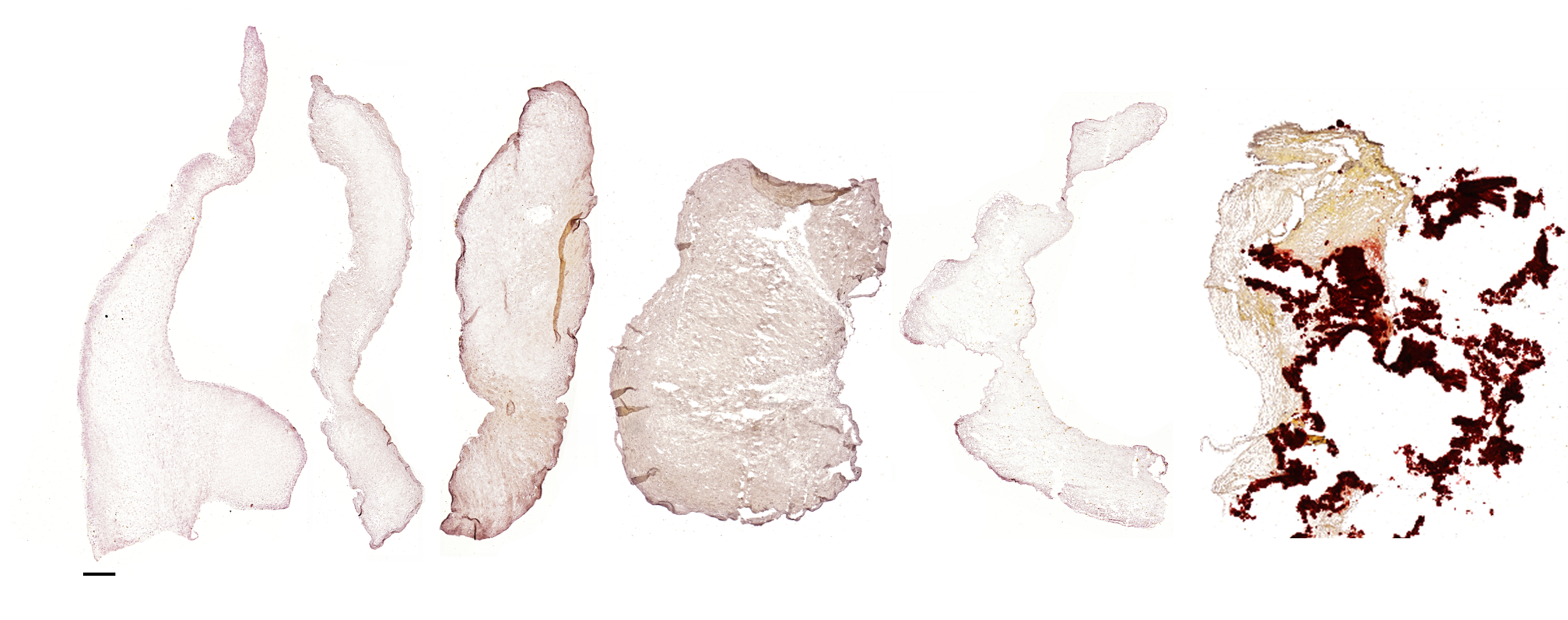

Supplement: Supplementary file 1 — Additional file 1: Supplementary Figure 1. AV tissue calcification after pulsatile dynamic vs. static tissue culture: pAVs were stained with Alizarin red to investigate valvular calcification at the beginning of the experiment and after 14 days of high and low flow dynamic, static and lysis incubation conditions. Calcified hAV was used after explantation as positive control. (left to right, n=3, scale bar: 200 µm). [file 13036_2023_377_MOESM1_ESM.tif]

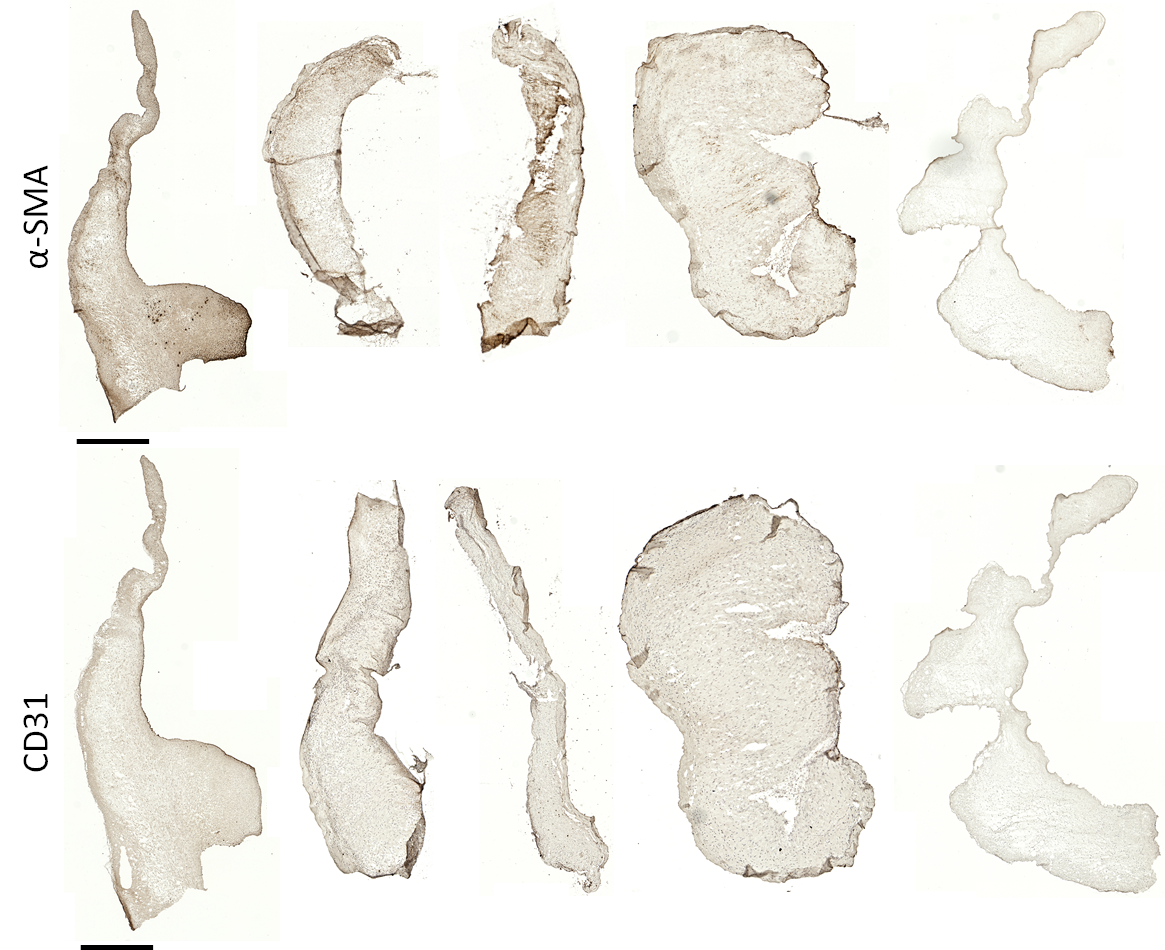

Supplement: Supplementary file 2 — Additional file 2: Supplementary Figure 2. Expression of α-SMA and CD31 in pAV tissue after pulsatile dynamic vs. static tissue culture: pAVs (high flow dynamic, low flow dynamic, static and death tissue conditions, 14 days; left to right, representative samples shown) were stained via immunohistochemistry to verify expression of the respective marker. No significant differences were detected for α-SMA expression. Rate of CD31 positive signal was significantly higher in statically incubated samples (shown in Figure 12; scale bar: 500 µm). [file 13036_2023_377_MOESM2_ESM.tif]
